# Effectiveness, Cost-effectiveness, and Cost-Utility of a Digital Alcohol Moderation Intervention for Cancer Survivors: Health Economic Evaluation and Outcomes of a Pragmatic Randomized Controlled Trial

**DOI:** 10.2196/30095

**Published:** 2022-02-01

**Authors:** Ajla Mujcic, Matthijs Blankers, Brigitte Boon, Anne H Berman, Heleen Riper, Margriet van Laar, Rutger Engels

**Affiliations:** 1 Erasmus School of Social and Behavioural Sciences Erasmus University Rotterdam Rotterdam Netherlands; 2 Trimbos Institute Utrecht Netherlands; 3 Department of Psychiatry Amsterdam University Medical Center, Location Amsterdam Medical Center University of Amsterdam Amsterdam Netherlands; 4 Department of Research Arkin Mental Health Care Amsterdam Netherlands; 5 Academy het Dorp Arnhem Netherlands; 6 Siza Arnhem Netherlands; 7 Tranzo Tilburg University Tilburg Netherlands; 8 Department of Psychology Uppsala University Uppsala Sweden; 9 Centre for Psychiatry Research Department of Clinical Neuroscience Karolinska Institute Stockholm Sweden; 10 Stockholm Health Care Services Stockholm Region Stockholm Sweden; 11 Section Clinical Psychology Amsterdam Public Health Research Institute Vrije Universiteit Amsterdam Amsterdam Netherlands; 12 Department of Psychiatry Medical University University of Turku Turku Finland; 13 Department of Psychiatry Amsterdam Public Health Research Institute Amsterdam University Medical Center Amsterdam Netherlands

**Keywords:** alcohol, brief interventions, cancer survivors, effectiveness, cost-effectiveness, eHealth, mobile phone

## Abstract

**Background:**

Alcohol moderation (AM) interventions may contribute to better treatment outcomes and the general well-being of cancer survivors.

**Objective:**

This study evaluates the effectiveness, cost-effectiveness, and cost-utility of MyCourse, a digital AM intervention, compared with a noninteractive digital information brochure for cancer survivors.

**Methods:**

A health economic evaluation alongside a pragmatic 2-arm parallel-group randomized controlled trial was conducted with follow-ups at 3, 6, and 12 months after randomization. The study was conducted on the web in the Netherlands from 2016 to 2019. Participants were adult 10-year cancer survivors drinking over the Dutch-recommended drinking guidelines (≤7 standard units [10 g of alcohol] per week) with the intention to moderate or quit drinking. Overall, 103 participants were randomized and analyzed: 53 (51.5%) in the MyCourse group and 50 (48.5%) in the control group. In the MyCourse group, participants had access to a newly developed, digital, minimally guided AM intervention, *MyCourse–Moderate Drinking*. The primary outcome was the self-reported number of standard drinks (10 g of ethanol) consumed in the past 7 days at the 6-month follow-up. The secondary outcome measures were alcohol-related problems as measured by the Alcohol Use Disorders Identification Test (AUDIT) and treatment satisfaction. For the health economic evaluation, health care costs, costs because of productivity losses, and intervention costs were assessed over a 12-month horizon.

**Results:**

Alcohol use at the 6-month follow-up decreased by 38% in the MyCourse group and by 33% in the control group. No difference in 7-day alcohol use was found between the groups (*B*=2.1, 95% CI −7.6 to 3.1; *P*=.22) at any of the follow-ups. AUDIT scores for alcohol-related problems decreased over time in both groups, showing no significant difference between the groups (Cohen *d*=0.3, 95% CI −0.1 to 0.6; *P*=.21). Intervention costs per participant were estimated at US $279 for the MyCourse group and US $74 for the control group. The mean societal costs were US $18,092 (SD 25,662) and US $23,496 (SD 34,327), respectively. The MyCourse group led to fewer gained quality-adjusted life years at lower societal costs in the cost-utility analysis. In the cost-effectiveness analysis, the MyCourse group led to a larger reduction in drinking units over time at lower societal costs (incremental cost-effectiveness ratio per reduced drink: US $ −1158, 95% CI −1609 to −781).

**Conclusions:**

At 6 months, alcohol use was reduced by approximately one-third in both groups, with no significant differences between the digital intervention MyCourse and a noninteractive web-based brochure. At 12 months, cost-effectiveness analyses showed that MyCourse led to a larger reduction in drinking units over time, at lower societal costs. The MyCourse group led to marginally fewer gained quality-adjusted life years, also at lower societal costs.

**Trial Registration:**

Netherlands Trial Register NTR6010; https://www.trialregister.nl/trial/5433

**International Registered Report Identifier (IRRID):**

RR2-10.1186/s12885-018-4206-z

## Introduction

Alcohol use is one of the main lifestyle factors influencing cancer development, and there is also evidence that it negatively affects the development of new malignancies [1], cancer treatment success [2], and mortality rates [3]. Therefore, it is recommended that cancer survivors quit or minimize alcohol use [4]. Currently, drinking rates among cancer survivors are comparable with drinking rates among the general population, with estimates that 5.1% of cancer survivors are heavy drinkers (>2 drinks per day for men; >1 drink per day for women) versus 6% of the general population [5].

Studies evaluating alcohol moderation (AM) interventions in cancer survivors are scarce. AM interventions offer support in reducing or quitting alcohol use and can range from brief face-to-face interventions by health care providers to smartphone app–based interventions. A 2018 review on smoking and alcohol cessation interventions in patients with head and neck cancer and oral dysplasia [6] found no randomized controlled trials (RCTs) evaluating AM interventions for head and neck cancer survivors, and neither did a recent meta-analysis on AM distance–based interventions for cancer survivors of all cancer types [7]. The latter review identified a few studies that incorporated AM as a module in a broader lifestyle program and found insufficient evidence of the interventions’ effectiveness on AM. A qualitative study assessed patients’ experiences with a face-to-face alcohol cessation program in bladder cancer survivors undergoing surgery; results indicated that major bladder surgery was a useful cue for motivating patients with cancer to reconsider the consequences of risky drinking, and the alcohol intervention was seen as a relevant offer around the time of surgery [8]. Facilitating access to AM interventions for cancer survivors via distance-based interventions, particularly digital ones, might be an effective and highly accessible means to provide the growing population of cancer survivors with AM support [9].

Studies among the general population have shown that brief face-to-face and digital interventions can be effective in reducing alcohol consumption. An individual patient data meta-analysis comparing guided and unguided low‐intensity internet interventions for AM found that participants in both types of interventions used on average 50 g less ethanol per week than the controls (5.02 standard units of 10 g of ethanol, 95% CI −7.57 to −2.48) [10]. A conventional meta-analysis evaluating brief AM interventions delivered in a primary care setting found that participants used on average 20 g (95% CI −28 to −12) less ethanol per week than the controls [11]. A meta-analysis of personalized digital interventions found similar results when comparing the interventions to nonintervention control groups (23 g less ethanol per week in participants receiving a digital intervention compared with no or minimal interventions, 95% CI 15-30 based on 41 studies) and found no difference in reduction of alcohol consumption in personalized digital interventions compared with face-to-face interventions, based on 5 studies [12].

Brief alcohol interventions in primary care settings have been found to be cost-effective for the general population [13]. Referral to a digital AM intervention was a cost-effective strategy in 3 European countries [14]. A game with tailored feedback on alcohol awareness was found to be cost-effective from the societal perspective in reducing the number of drinks in subgroups (older age and lower educational level) of adolescents [15]. We found no studies on the cost-effectiveness of digital AM interventions for cancer survivors, but cost-effectiveness is a key element in the knowledge base needed for policy decisions regarding implementation and financing of digital interventions [16,17]. It is unknown what results in effectiveness and cost-effectiveness are to be obtained from a digital AM intervention that is tailored to cancer survivors, as cancer survivors have increased feelings of distress and symptoms of anxiety and depression [18,19], and they could have additional benefits of AM (eg, in terms of treatment outcomes) [2].

Therefore, it was deemed necessary to evaluate both the effectiveness and cost-effectiveness of a minimally-guided digital intervention aimed at supporting cancer survivors to moderate their alcohol use: *MyCourse–Moderate Drinking* (in Dutch: *MijnKoers–Minderen met Drinken*). The development process and a detailed intervention description are provided elsewhere [20]. In this study, we aim to answer the following research questions: (1) Is the digital, minimally guided AM intervention *MyCourse–Moderate Drinking* more effective than a digital AM brochure to moderate alcohol use? (2) From a societal perspective, is the digital, minimally-guided AM intervention *MyCourse–Moderate Drinking* more cost-effective than a web-based AM brochure in terms of incremental costs per reduced weekly drink and incremental costs per quality-adjusted life year (QALY) gained?

We expect the MyCourse intervention to be both more effective and more cost-effective than a web-based brochure on AM.

## Methods

### Design

In a 2-arm, individually randomized RCT conducted in the Netherlands between 2016 and 2019, the effectiveness and cost-effectiveness of the *MyCourse–Moderate Drinking* intervention for cancer survivors was evaluated. The first inclusion was on November 28, 2016, and the last inclusion was on September 3, 2018. The last follow-up measurement was collected on September 30, 2019. The study was prospectively registered in the Netherlands Trial Register (NTR6010). The planned inclusion period was extended by several months to recruit as many participants as possible. An extensive description of the study protocol has been provided in the study by Mujcic et al [20]. This study was part of a set of 2 separate RCTs on digital interventions for AM and smoking cessation in cancer survivors. The results of the RCT on the smoking cessation intervention (*MyCourse–Quit Smoking*) will be published separately. Ethical approval was obtained from an accredited medical research and ethics committee in the Netherlands (Toetsingscommissie Wetenschappelijk Onderzoek Rotterdam NL55921.101.16).

### Participants and Recruitment

**Figure 1:**
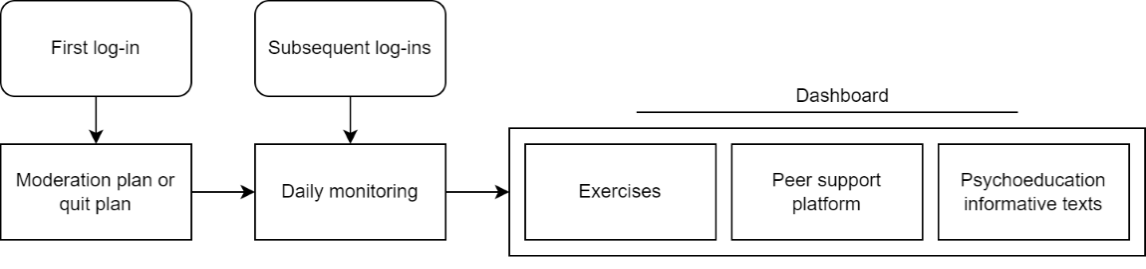
Intervention flowchart (adapted from Mujcic et al [20]).

Participants could find out about the study and apply for participation via a web-based screening questionnaire on a dedicated website that was created for the study. Participants were eligible if they were aged ≥18 years, had been diagnosed with any form of cancer in the past 10 years, had a PC or laptop and internet connection at home, had the ability and intention to participate in the study for 12 months, used alcohol more than recommended by Dutch guidelines (operationalized as drinking >7 European standard units of alcohol [70 g of ethanol] per week), and had the intention to reduce their alcohol use. The exclusion criteria were insufficient mastery of the Dutch language, pregnancy or self-reported suicidal ideation, acute psychosis, severe alcohol dependence, dementia, or severe depression at the time of screening. The same screening questionnaire procedure was used for both this trial and the similar parallel trial evaluating a smoking cessation intervention [20]. Some people were eligible for both trials; if so, they were allowed to participate in only one of the trials, based on their own choice (Figure 1 [20]).

Both web-based and offline strategies were used for the recruitment of participants. Targeted web-based advertisements on social and other media, and on search engines referred those interested to the website and the web-based screening questionnaire. Patient organizations, oncology departments in hospitals, and meeting centers for cancer survivors were contacted and offered promotional material (flyers and posters) to help refer cancer survivors to the website.

### Procedure

After filling out the screening questionnaire on the study website, applicants were informed by a computer-generated email about their eligibility for study participation. Those eligible were sent an invitation email containing all relevant patient information, the informed consent form, and a link to register. Eligible cancer survivors had up to 30 days to decide about their participation, and during this period, they could contact the research team or an independent physician with questions. After they had digitally signed the informed consent form, they were sent the baseline questionnaire. Immediately after completion of the baseline measurement, participants were automatically allocated to either the MyCourse or the control group arm in a 1:1 ratio through adaptive randomization (minimization of baseline imbalances with regard to age, sex, and education level) through a server-sided hypertext preprocessor script using a Mersenne twister random number generator. Participants received an email confirming their allocation and containing their username and instructions on how to log on. They were not blinded to study condition allocation (the participants were not explicitly informed about their allocation, but recruitment material included a video showing interactive elements of MyCourse, making it plausible that the participants knew they were not allocated to the experimental condition. Thus, we cannot consider the participants blinded). At 3, 6, and 12 months after randomization, the participants received a link to the web-based questionnaire via email. The nonresponders received up to 3 reminder emails, and in case of continued nonresponse they were contacted by telephone. For each completed follow-up assessment, they were reimbursed with €25 (approximately US $30). As this was a pragmatic RCT, patients in both groups were not asked to refrain from using additional support if they wanted to.

### MyCourse Group

*MyCourse–Moderate Drinking* is a newly developed, minimally-guided, digital intervention aimed at supporting AM in cancer survivors. It is based on well-established therapeutic approaches: motivational interviewing, cognitive behavioral therapy, and acceptance and commitment therapy, as well as a Dutch digital AM intervention previously found to be effective in the general population [21]. Throughout the development process, cancer survivors and professional experts in eHealth, oncology, and substance use disorders were involved through a series of interviews and focus groups. The intervention was accessible through PC, tablet, and smartphone. At first log-in, the participants were guided by website prompts in either setting up a quit plan or a moderation plan, including a quit date or moderation date, after which they would gain access to several exercises, a web-based diary for self-monitoring of alcohol use and contextual cues, and a peer support platform (See Figure 1 adapted from Mujcic et al [20] and Multimedia Appendix 1 [22]). MyCourse could be used by the participants whenever they chose to, but they were encouraged to log in daily for at least 4 weeks. The intervention and its development process have been extensively described elsewhere [20].

### Control Group

The control group consisted of a noninteractive web-based static information brochure on the risks of (increased) alcohol use and tips on how to moderate or quit drinking. It was accessible to the participants at any time by logging into the website. The brochure contained both general information on AM and information specifically relevant to cancer survivors. However, no interactive elements of the MyCourse condition were present, and the participants did not receive reminders.

### Additional Support

Both groups were provided with the contact details of the national AM information line (in Dutch: *Alcoholinfo-lijn*), which could help refer participants to additional support if they deemed the received intervention to be insufficient. At the end of the study, at 12 months after randomization, the participants in the control group received access to the digital intervention, *MyCourse–Moderate Drinking,* which was offered to the MyCourse group.

### Measures

#### Baseline

At baseline, we assessed the sociodemographic characteristics and type of cancer. Alcohol use was assessed using Timeline Followback (TLFB) self-reports [23] (number of standard drinks consumed in the past 7 days, ie, 7-day alcohol use). Problematic alcohol use was assessed using the 10-item Alcohol Use Disorders Identification Test (AUDIT) questionnaire. In participants reporting smoking, we assessed tobacco use with TLFB self-reports and nicotine dependence using the 6-item Fagerstrom Nicotine Dependence Test questionnaire [24]. Socially desirable answering tendencies, which may have affected the reliability of the self-reported questionnaire data, were assessed using the Marlowe-Crowne Social Desirability Scale (MCSDS) [25]. We used the 5-level EuroQol 5 Dimension (EQ-5D-5L) [26] measure to assess QALYs. The Medical Outcomes Study Short Form [27] was used to calculate the Short Form 6-dimension (SF-6D) quality of life measure using the Brazier algorithm [28].

#### Follow-up Measurements

At all follow-up measurements, we assessed alcohol use with TLFB self-reports, quality of life using the EQ-5D-5L and the Medical Outcomes Study Short Form, productivity and health care costs, and use of other AM support. Intervention use variables (eg, number of log-ins and use of major content elements) were collected automatically. The AUDIT questionnaire was administered at the 6- and 12-month follow-ups. At the 3-month follow-up, treatment satisfaction was assessed using a Dutch translation of the German adapted Client Satisfaction Questionnaire (Fragebogen zur Messung der Patientenzufriedenheit; Patient Satisfaction Questionnaire [ZUF-8]) [29]. The use of additional support for AM was retrospectively assessed at follow-up.

#### Primary and Secondary Outcome Measures

The primary outcome measure was 7-day alcohol use (number of standard drinks; 1 standard drink=10 g of ethanol) at the 6-month follow-up measured by TLFB self-reports. The 6-month assessment was the primary end point, as the studies that formed the basis of our power analysis were based on outcomes at the 6-month follow-up and it is a common end point in alcohol trials [30]. Those who reported no drinking at all in the past 7 days were considered abstinent (score: 0/1). Secondary outcome measures were AUDIT problematic alcohol use (score: 0-40), ZUF-8 treatment satisfaction (score: 8-32), EQ-5D-5L quality of life (score: 0-1), health care costs, and productivity loss.

#### Costs

Costs were calculated from a societal perspective for the index year 2019. Intervention costs included intervention depreciation costs, costs for hosting the website, technical support, and recruitment costs (which consisted of both advertising costs in web-based and offline media as well as printing costs of promotional material). Recruitment costs were included as they were considered an essential part of the MyCourse and control groups. Health care costs were calculated by multiplying the number of reported contacts with a health care professional with the standard unit cost prices for the Netherlands [31]. Health service costs stemmed from contacts with specialized somatic and mental health care, plus the patients’ out-of-pocket costs for home care, but travel costs were not included because, in both groups, the interventions were delivered over the internet. Other health care costs included appointments for physiotherapy, alternative medicine, and social work. Medication costs were calculated by multiplying the reported dose of a drug with its unit cost price [32].

Productivity loss included costs from absenteeism and presenteeism, calculated according to the friction cost method, meaning productivity losses were limited to a maximum of 85 days, after which production losses cease to exist because the sick employee has been replaced by another and calculated using an elasticity factor of 0.8 as there is not a strict 1:1 relation between days not worked and productivity losses. Cost data related to health care use and productivity loss were assessed using the Trimbos/Institute for Medical Technology Assessment questionnaire for costs associated with psychiatric illness [33] at all follow-up assessments. Cumulative societal costs over the entire follow-up period of 12 months were calculated from the sum of health care costs and productivity losses. Costs were converted from euros to US dollars using purchasing power parities for the reference year 2019. No discount rate was applied as the follow-up period was 12 months.

### Sample Size

The sample size was based on conventional levels of statistical significance (α≤.05). On the basis of the average of 2 previous RCTs on very similar self-help interventions in the Netherlands versus a control group [21,30,34], a Cohen *d* effect size of 0.40 was expected. Using the power calculation package pwr for R 3.0.1 (R Foundation for Statistical Computing) [35], we calculated that a sample size of 2 × 57 participants in the case of 1-sided testing led to a power of 0.77 or a power of 0.66 in the case of 2-sided testing. The choice for 1-sided testing was discussed in the previously published protocol paper [20].

### Statistical Analyses

#### Imputation of Missing Data

Except for the ZUF-8 (treatment satisfaction) questionnaire, all primary and secondary outcome measures were analyzed in accordance with the intention-to-treat principle. To that end, missing data for primary and secondary outcome measures, and costs, were multiple-imputed using the predictive mean matching method from the mice package in R [36]. For each missing observation, 50 imputations were created. The responses to the ZUF-8 questionnaire were not imputed.

#### Effect Evaluation

Alcohol use in the past 7 days (count data 0, 1...,N) was analyzed using robust estimation of generalized linear mixed models from the robustlmm package in R [37], as the data did not fit well into any of the commonly supported distributions. Imputation of missing values before running a generalized linear mixed model allowed us to consider all variables that could have affected the dropout. Covariates in the model were the minimized variables (gender, age, and education) and the MCSDS (to statistically account for any social desirability of responses). Model estimates, Cohen *d*, 95% CIs, and *P* values were reported. Differences over time and between the groups on AUDIT problematic alcohol use and ZUF-8 patient satisfaction scores were analyzed using a linear mixed model in the lme4 package in R [38]. We used 1-sided testing and an α of .05 as described in the study protocol [20].

#### Cost-effectiveness Analyses

An economic evaluation was conducted alongside the RCT in concordance with the Consolidated Health Economic Evaluation Reporting Standards Statement [39] and following the approach by Drummond et al [40]. QALYs over the entire follow-up period were computed using the Dutch tariff (utility weights) [41] and the area under the curve method. The incremental cost-effectiveness ratio (ICER) was calculated as follows:

ICER = (C_1_ − C_0_) / (E_1_ − E_0_) **(1)**

where C refers to costs, E refers to effects, and the subscripts 0 and 1 refer to the MyCourse and control arms, respectively. We generated 2500 nonparametric bootstrapped samples and plotted the corresponding incremental costs and incremental effects on a cost-effectiveness plane. Both ICER per QALY and ICER per reduced weekly drink were calculated from the following 4 perspectives: societal, health care, productivity loss, and intervention cost only. Cost-effectiveness acceptability curves (CEACs) were also drawn to assess the likelihood that the experimental intervention will be deemed cost-effective given a series of willingness-to-pay ceilings.

#### Sensitivity Analyses

The robust regression on the mice-imputed data was the main analysis. We conducted several sensitivity analyses to assess effectiveness and cost-effectiveness using QALYs based on the SF-6D (instead of the EQ-5D-5L), imputation using the Amelia II package instead of the mice package, Winsorization of costs, and different statistical models (Multimedia Appendix 1).

## Results

### Sample Characteristics

**Figure 2:**
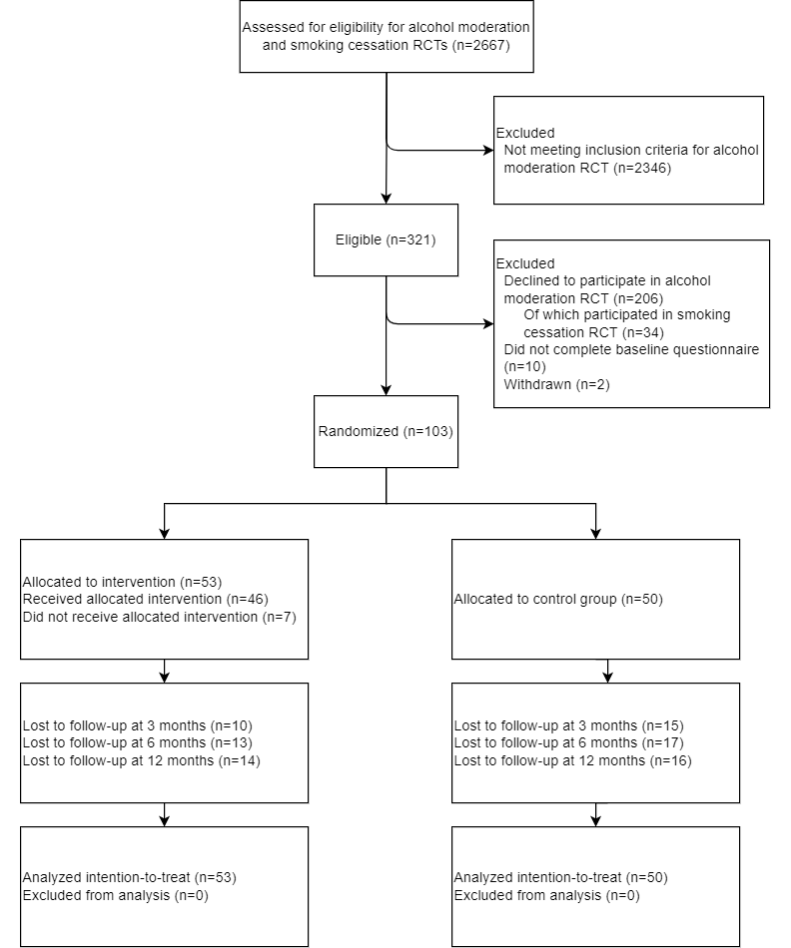
CONSORT (Consolidated Standards of Reporting Trials) flowchart. RCT: randomized controlled trial.

The participant flow and retention rates are shown in Figure 2. Of the 2346 ineligible people, 1684 (71.78%) had had no diagnosis of cancer in the past 10 years. A total of 321 cancer survivors were eligible for participation in the study, of whom 206 (64.2%) declined to participate, and 34 (10.6%) participated in the study on smoking cessation instead. Of 115, 10 (8.7%) cancer survivors did not complete the baseline questionnaire and were therefore not randomized, and 2 (1.7%) cancer survivors withdrew during the course of the study. This resulted in a study sample of 103 participants; of whom, 53 (51.5%) were randomized into the experimental MyCourse group and 50 (48.5%) were randomized into the control group. Table 1 presents the sociodemographic and other characteristics of the sample. In summary, the mean age of the participants was 54.6 (SD 11) years, 16.5% (17/103) were men, most were married or living with a significant other (70/103, 68%), and 31.3% (32/103) had a middle or low educational level. Breast cancer was the most frequently reported type of cancer (65/103, 63.1%). Problematic alcohol use as measured by the AUDIT was significantly higher in the MyCourse group than in the control group as calculated using the Welch 2-sample *t* test (2-tailed) for continuous variables (t_100.9_=2.03; *P*=.02). No difference was found in the proportion of missing data between the groups (*χ^2^*_1_=2.5 *P*=.11; see Multimedia Appendix 2 for details). Data were missing because of loss to follow-up (participants who did not respond after several reminders by email and telephone).

**Table 1:** Baseline characteristics^a^.

Characteristic		MyCourse (n=53)	Control (n=50)	Total (N=103)
**Gender, n (%)**				
	Women	46 (87)	40 (80)	86 (83)
	Men	7 (13)	10 (20)	17 (17)
Age (years), mean (SD)		54.5 (12.1)	54.6 (9.9)	54.6 (11)
**Education, n (%)**				
	Higher level	34 (64)	37 (74)	71 (69)
	Midlevel	11 (21)	11 (22)	22 (21)
	Lower level	8 (15)	2 (4)	10 (10)
**Marital status, n (%)**				
	Married or living together	34 (64)	36 (72)	70 (68)
	Unmarried or living alone	6 (11)	8 (16)	14 (14)
	Divorced	10 (19)	4 (8)	14 (14)
	Widowed	3 (6)	2 (4)	5 (5)
**Drinking behavior, mean (SD)**				
	Number of drinks in past 7 days	26.8 (19.0)	20.7 (14.7)	23.8 (17.2)
	AUDIT^b^	14.5 (6.0)	12.2 (5.4)	13.3 (5.8)
**Smoking behavior**				
	Smoked in last month, n (%)	10 (19)	6 (12)	16 (16)
	Number of cigarettes in past 7 days among smokers, mean (SD)	87.9 (52.6)	81.6 (68.5)	85.3 (56.8)
	Nicotine dependence, mean (SD)	0.6 (1.7)	0.3 (1.3)	0.5 (1.5)
**Cancer diagnosis, n (%)**				
	Breast	38 (72)	27 (54)	65 (63)
	Uterus	4 (8)	2 (4)	6 (6)
	Head and neck	1 (2)	4 (8)	5 (5)
	Colon	2 (4)	3 (6)	5 (5)
	Lung	1 (2)	2 (4)	3 (3)
	Other (including bladder, lymphatic, melanoma, skin, and prostate)	7 (13)	12 (24)	19 (18)

^a^Percentages may not add up to 100 because of rounding.

^b^AUDIT: Alcohol Use Disorders Identification Test.

### Treatment Uptake and Satisfaction

Overall, patients were most satisfied in the MyCourse group (Cohen *d*=0.81; *t*_61.5_=3.42; *P*<.001; see Multimedia Appendix 2 for the mean scores). Most participants logged in at least once (46/53, 87%). The average number of times the participants logged in was 31.4 (SD 50.5), with a median of 8 (range 0-254). For those who logged in at least once, the period between the first and last log-in was on average 105.6 (SD 125.6) days with a median of 45 days. There was little use of AM support besides MyCourse; no support was reported most often (control group: 26/50, 52%; MyCourse group: 26/53, 49%) and some connected with others who were also moderating their drinking (control group: 4/50, 8%; MyCourse group: 5/53, 9%). Of 103 participants, only 1 (0.9%) reported having had contact with a health care professional about AM.

### Incremental Effects

#### Primary Outcome

Despite the randomization, there was an apparent, although nonsignificant, difference between the groups in baseline alcohol use (Table 1). The number of drinks consumed in the past week at the 6-month follow-up decreased by 38% in the MyCourse group and by 33% in the control group and even more at the 12-month follow-up—by 48% in the MyCourse group and 38% in the control group (Table 2). No difference in 7-day alcohol use was found between the groups (unstandardized regression coefficient, *B*=−2.1, 95% CI −7.6 to 3.1; *P*=.22; Table 3) at 6 months—or at any of the other follow-up assessments—when controlling for MCSDS score, baseline alcohol use, gender, age, and education.

**Table 2:** Drinking behavior outcomes at the 3-, 6-, and 12-month follow-ups (N=103)^a^.

Variable		MyCourse (n=53)	Control (n=50)
**Number of drinks in past 7 days, mean (SD)^b^**			
	Baseline	26.8 (19.0)	20.7 (14.7)
	3-month follow-up	17.3 (15.8)	15.1 (11.9)
	6-month follow-up	16.6 (15.2)	13.8 (11.4)
	12-month follow-up	13.9 (11.0)	12.9 (10.7)
**Change in number of drinks in past 7 days,** **mean (SD)^c^**			
	3-month follow-up	−8.5 (12.0)	−5.2 (13.5)
	6-month follow-up	−9.4 (15.0)	−6.4 (16.4)
	12-month follow-up	−12.1 (16.3)	−7.4 (13.3)
**AUDIT^d^, mean (SD)**			
	Baseline	14.5 (6.0)	12.2 (5.4)
	6-month follow-up	11.3 (6.2)	9.9 (5.1)
	12-month follow-up	10.0 (6.0)	9.3 (5.1)
**Abstinence, n (%)**			
	3-month follow-up	6 (11)	7 (14)
	6-month follow-up	6 (11)	8 (16)
	12-month follow-up	5 (10)	7 (14)

^a^Missing data were imputed.

^b^The number of drinks per day was maximized at 11 units in the follow-up measurements for the imputation of missing data, meaning that 77 was the maximum number of drinks in the past 7 days.

^c^Mean number of drinks at follow-up minus the mean number of drinks at baseline.

^d^AUDIT: Alcohol Use Disorders Identification Test.

**Table 3:** Treatment effects on drinking behavior at the 3-, 6-, and 12-month follow-ups^a^.

Outcome measure		Treatment effect		
		*B*_adjusted_ (SE; 95% CI)	*P* value	Cohen *d* (95% CI)
**Number of drinks in past 7 days^b^**				
	3-month follow-up	−3.2 (2.6; −8.5 to 1.9)	.11	N/A^c^
	6-month follow-up	−2.1 (2.7; −7.6 to 3.1)	.22	N/A
	12-month follow-up	−3.7 (2.7; −8.9 to 1.6)	.09	N/A
**AUDIT^d^**				
	6-month follow-up	−0.9 (1.0)^e^	.21	0.3 (−0.1 to 0.6)
	12-month follow-up	−1.6 (1.0)^e^	.06	0.1 (−0.2 to 0.5)

^a^Missing data were imputed.

^b^Adjusted coefficients are based on a robust regression mixed model with random intercept and fixed slope in which the outcome measure at follow-up is regressed upon baseline number of drinks, covariates, and group.

^c^N/A: not applicable.

^d^AUDIT: Alcohol Use Disorders Identification Test (adjusted coefficients are based on a linear mixed model with random intercept and fixed slope in which the outcome measure at follow-up is regressed upon baseline number of drinks, covariates, and group).

^e^95% CI value is not available.

#### Secondary Outcomes

**Figure 3:**
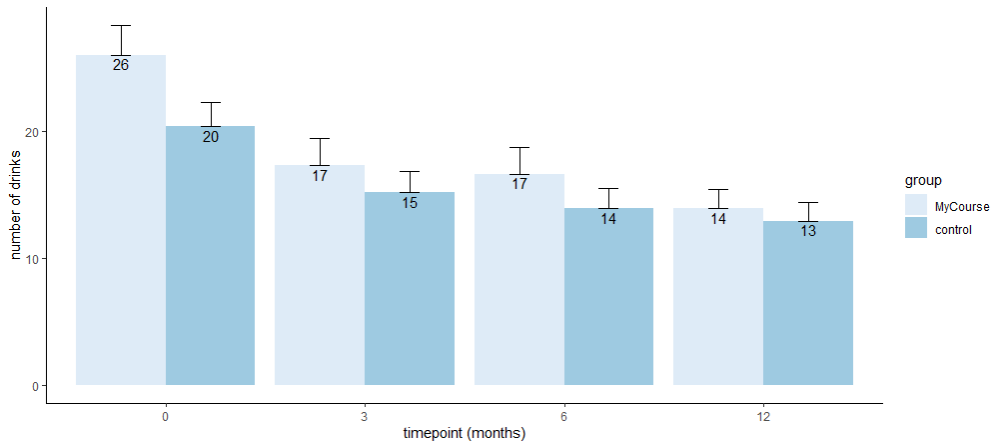
Mean number of drinks in the past 7 days in both groups at baseline and during the course of the study, including SEs.

AUDIT scores decreased over time in both groups (Table 2 and Figure 3), but there was no difference between the groups (Cohen *d*=0.3, 95% CI −0.1 to 0.6; *P*=.21; Table 3) at 6 months. The mean EQ-5D-5L QALYs score in the MyCourse group was 0.82 (SD 0.12) and in the control group 0.84 (SD 0.10). There was no significant effect of the treatment on the quality of life based on EQ-5D-5L scores (*B*_adjusted_=0.003, SE 0.01; *P*=.39).

### Incremental Costs

Table 4 presents the costs per group and the incremental costs (cost difference between the MyCourse and control groups) per cost item. The intervention was costed at US $279 per participant in the MyCourse group and US $74 per participant in the control group. The average health care costs accumulated over the full 12-month follow-up time were US $7840 (SD 11,767) per participant in the MyCourse group and US $8233 (SD 15,077) per participant in the control group, and the incremental health care costs were US $−393. Costs owing to productivity losses were mainly driven by absenteeism: US $9532 (SD 19,389) per participant in the MyCourse group and US $14,799 (SD 23,364) per participant in the control group, with high within-group variance. Incremental productivity costs per participant were on average US $−5217 (SD 26,378). The average cumulative societal costs were US $5404 (SD 42,859) lower in the MyCourse group compared with the control group.

**Table 4:** Mean cumulative costs (in US $) by group and incremental costs (N=103).

Cost item		MyCourse (n=53), mean (SD)	Control (n=50), mean (SD)	Incremental costs^a^ (n=53), mean (SD)
**Health care costs**		7840 (11,767)	8233 (15,077)	−393 (19,125)
	Specialized somatic	3819 (5772)	3627 (6463)	192 (8665)
	Specialized psychiatric	1209 (3878)	688 (1906)	521 (4321)
	Patient and family costs	953 (5517)	178 (1811)	775 (5807)
	Other	907 (1142)	1126 (1434)	−219 (1833)
	Medication	953 (5479)	2613 (10,521)	−1660 (11,862)
**Productivity loss**		9972 (1934)	15,189 (26,307)	−5217 (26,378)
	Presenteeism	153 (319)	210 (408)	−57 (518)
	Absenteeism	9532 (19,389)	14,799 (26,364)	−5267 (32,726)
	Unpaid work	452 (1049)	474 (1007)	−22 (1454)
Intervention costs		279 (0)	74 (0)	205 (0)
Total societal costs		18,092 (25,662)	23,496 (34,327)	−5404 (42,859)

^a^Costs in the MyCourse group minus costs in the control group.

### Cost-Utility

**Figure 4:**
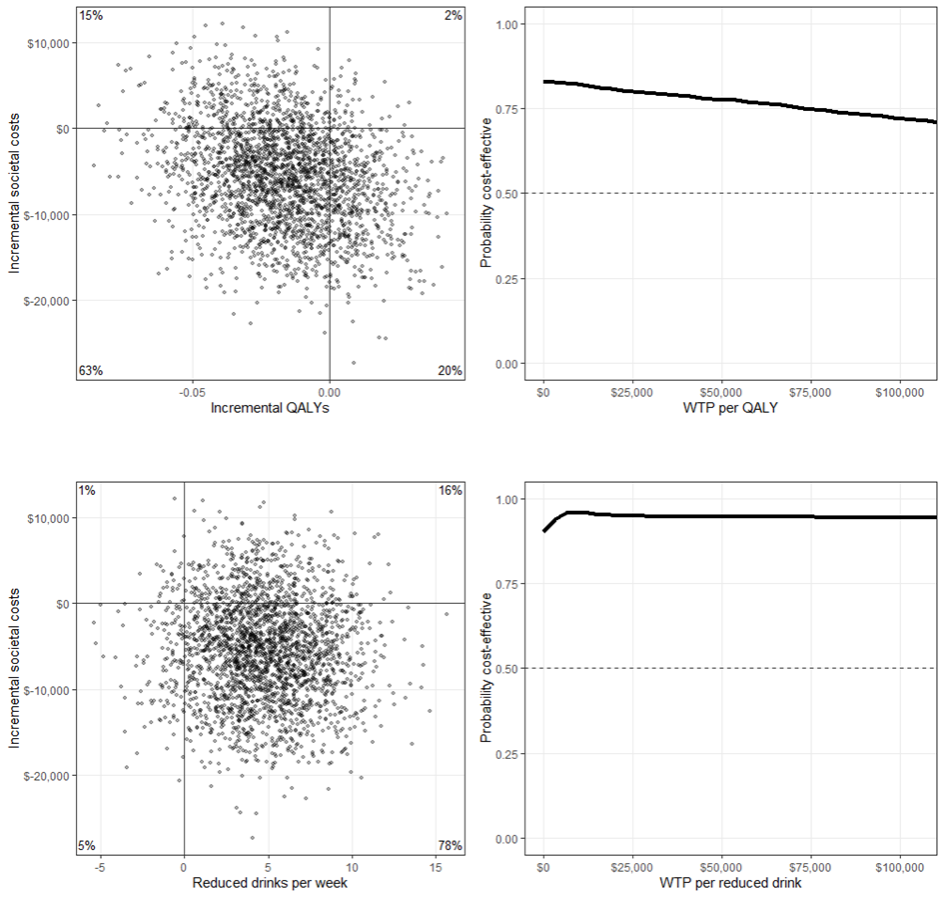
Cost-effectiveness planes and cost-effectiveness acceptability curves in US $. QALY: quality-adjusted life year; WTP: willingness to pay.

With QALY as the outcome, the ICER was US $314,606 (95% CI 186,201-553,552). The cost-effectiveness plane (Figure 4) shows that there is a 63% chance that the MyCourse intervention will lead to fewer QALYs gained at lower societal costs and a 15% chance that it will lead to more QALYs gained at lower societal costs. The relatively high ICERs are mostly a result of a difference in productivity costs (Table 4) and a very small differential effect on QALYs.

Assuming an intervention-cost-only perspective, the ICER per QALY gained became negative (US $−11,930; 95% CI −18,440 to −8912), indicating that intervention costs were higher and the QALY gains were lower in the MyCourse group compared with the control group.

### Cost-effectiveness

The MyCourse group reduced their number of weekly drinks more on average (mean 12.1, SD 16.3 drinks) than the control group (mean 7.4, SD 13.3 drinks) over a 12-month period and at lower societal costs. ICER per reduced drink was calculated at US $−1158 (95% CI −1609 to −781), indicating that compared with the control group each additional reduced drink in the MyCourse group was associated with a societal cost reduction. There is a 78% chance that the MyCourse intervention will lead to more weekly reduced drinks at lower societal costs and a 16% chance that it will lead to more weekly reduced drinks at higher costs (Figure 4). MyCourse will be preferred over the control group at any willingness-to-pay level (see Figure 4 for the CEAC curve). Assuming an intervention-cost-only perspective, a reduction of 1 additional weekly drink would cost an additional US $44 (95% CI 38-53) in the MyCourse group compared with the control group. Table 5 shows a breakdown by perspective.

**Table 5:** Incremental cost-effectiveness ratios between baseline and the 12-month follow-up^a^.

Perspective	Incremental costs per QALY^b^ (US $)	Incremental costs per reduced drink (US $)
	Value, mean (95% CI)	Value, mean (95% CI)
Health care	22,859 (−18,584 to 78,705)	−84 (−242 to 74)
Productivity loss	303,677 (198,917 to 516,624)	−1118 (−1497 to −823)
Intervention cost only	−11,930 (−18,440 to −8912)	44 (38 to 53)
Societal	314,606 (186,201 to 553,552)	−1158 (−1609 to −781)

^a^The incremental cost-effectiveness ratios were calculated as follows: (C_1_–C_0_) / (E_1_–E_0_), where C refers to costs, E refers to effects, and the subscripts 0 and 1 refer to the experimental and control arms, respectively.

^b^QALY: quality-adjusted life year (as measured by the 5-level EuroQol 5 Dimension).

### Sensitivity Analyses

Sensitivity analyses of the Amelia II-imputed data (*B*_adjusted_=2.1, SE 1.8; *P*=.12) and completers-only data (*B*_adjusted_=1.7, SE 1.4; *P*=.12) as well as a negative binomial mixed model on the mice-imputed data (incidence rate ratio=1.05, 95% CI 0.79-1.4; *P*=.38) corroborated the main findings and showed no effect of treatment on the number of drinks in the past week. All sensitivity analyses showed a decrease in alcohol use in both groups. Because of the apparent, although nonsignificant difference, between the groups in baseline alcohol use, we also modeled the individual change scores in alcohol use in a robust regression. Although the average reduction in alcohol use at 6 and 12 months was larger in the MyCourse group, the difference was not statistically significant, yielding the same results (*B*_adjusted_=−2.1, SE 1.8; *P*=.12; Table 2). When QALYs were based on SF-6D scores, results of the economic evaluation remained similar. When Winsorization of extreme costs was applied at the 95th percentile, the cost-effectiveness planes and CEAC curves remained similar (Multimedia Appendix 3); however, ICER per EQ-5D-5L QALY was US $118,287 (95% CI 51,324-235,817), and ICER per reduced drink became less extreme (US $−435, 95% CI −680 to −219). Overall, the sensitivity analyses attested to the robustness of the findings in the main analysis.

## Discussion

### Principal Findings

We evaluated the effectiveness and cost-effectiveness of *MyCourse–Moderate Drinking*, a digital AM intervention for cancer survivors versus a web-based noninteractive information brochure. At the 6-month follow-up, the number of drinks in the past 7 days was reduced by 38% in the MyCourse group (mean −9.4, SD 15.0 standard units) and by 33% in the control group (mean −6.4, SD 16.4 standard units) and even further at the 12-month follow-up (MyCourse group: mean −12.1, SD 16.3 standard units; control group: mean −7.4, SD 13.3 standard units). No significant difference in 7-day alcohol use was found between the groups at any of the follow-up points. AUDIT scores decreased over time in both groups, but there was no statistically significant difference between the MyCourse group and the control group. Importantly, the participants were more satisfied in the MyCourse group.

In the cost-effectiveness analyses, the MyCourse group led to fewer QALYs and more reduced drinks, both at lower societal costs. Thus, MyCourse has shown to be more effective and cost-saving for number of reduced drinks.

From a societal perspective, the MyCourse group gained fewer QALYs at lower societal costs. The MyCourse intervention itself was associated with higher intervention costs than the noninteractive information brochure. Both ICERs reflected only marginally higher QALY gains in the control group. This study did not find any effect of MyCourse on QALYs. It could be hypothesized that a longer follow-up period would have been necessary for improvements in quality of life to take place in a population of cancer survivors, as their quality of life may be more directly influenced by factors pertaining to the cancer diagnosis (eg, invasiveness of cancer treatment, disease stage, cancer-related physical symptoms, and comorbidities) [42]. Therefore, we conclude that the MyCourse intervention seems more economically sustainable from a societal perspective than the noninteractive information brochure in reducing the number of drinks over a 12-month time horizon, whereas, to find evidence of possible cost-utility of a digital AM intervention, a longer follow-up period might be needed.

The difference in baseline alcohol use might be a possible explanation for the seemingly different conclusions between the incremental effect analysis and the cost-effectiveness analysis on the greater reduction of 7-day alcohol use in the MyCourse group. Even though the participants were randomized, at baseline, the participants in the MyCourse group had higher AUDIT scores and consumed more drinks on average than the participants in the control group (although the difference between the groups was not significant). At the 3-, 6-, and 12-month follow-ups, there was no significant difference in the number of drinks consumed in the past week or the AUDIT scores between the 2 groups. Thus, the larger nominal reduction in the number of drinks in the MyCourse group does not reflect a difference in the number of drinks at any of the follow-up assessments but rather a difference at baseline. This baseline difference translates differently in the incremental effect analysis, assessing differences at discrete time points, compared with the cost-effectiveness analysis, assessing differences over a period (12 months). It is also possible that, because of insufficient power, no significant difference was found in the incremental effect analysis, whereas, in the cost-effectiveness analysis, this was of lesser influence.

### Wider Context

Meta-analyses of brief AM interventions [11] and AM internet interventions [10] among the general population have found that alcohol use in the past 7 days in the intervention group was reduced by approximately 20-50 g more than in the control group. Although this study did not find a significant effect on the AM rates of the MyCourse intervention over the control condition, we did find considerable reductions of approximately 70 g of ethanol at the 12-month follow-up in the control group and 120 g in the MyCourse group. These increasing reductions at longer follow-up assessments were also found in a previous digital AM intervention study [43]. In line with limited previous studies on brief and digital AM interventions [13-15], this study showed that a digital AM intervention can be cost-effective among cancer survivors. Unfortunately, because of a lack of literature, no comparisons could be made to other dedicated AM interventions for cancer survivors. A study on a telephone-counseling, combined alcohol, smoking, and depression intervention for head and neck cancer survivors also found a decrease in AUDIT scores after 6 months in both groups and no differential effect between the experimental intervention and the control group receiving only the nurse-delivered, face-to-face, 45-minute assessment, including a handout with referrals for further care (which the experimental group received as well) [44].

The lack of difference in alcohol use in the past 7 days between the MyCourse group and the control group might be due to several study aspects. It is also possible that because of the inclusion criterion of having the intention to reduce one’s alcohol use, participants in both groups were highly motivated to change their alcohol use and that this obscured any effect of the MyCourse group. In addition, during this study, great efforts were made to recruit participants. At the time of recruitment in 2016-2018, AM discussion and support for cancer survivors was not well-implemented in many oncology settings; therefore, the researchers invested in informing oncology department staff on the importance and benefits of addressing AM in cancer survivors. Recruitment efforts were not only aimed at professionals; a dedicated website and a social media campaign were also in place, aiming to inform cancer survivors about the short-term benefits of AM after a cancer diagnosis while emphasizing an accepting tone to reduce possible feelings of guilt and ultimately guiding survivors to participate in the study. These recruitment efforts alone might have served as an intervention by focusing attention on AM. For cancer survivors not yet considering AM, the first step would be to address the knowledge gap on the adverse health effects of alcohol [45,46].

Second, the assessment load in this study was substantial, and the participants received multiple reminder emails and telephone calls from the researchers to fill out the survey at the respective follow-up measurement waves. Although these calls were kept as short as possible, some participants might have experienced them as part of the intervention; thus, possibly contributing to an intervention effect and making participants think regularly about their drinking behavior and feel supported [47,48]. This minimal guidance could thus have increased the AM rates in the control group. Future research should evaluate whether addressing AM in an accepting manner and with multiple repeated short reminders can encourage AM in cancer survivors.

It is possible that a true effect was not found in this study because the sample size was smaller than intended (103 instead of 114 participants). It is unlikely that the use of additional support explains the reduction of alcohol use in the control group, as very few people used any additional support. Low use of additional support was also found in a previous Dutch study on digital AM support [49]. Although the control group was as effective as the MyCourse group in reducing alcohol use in the incremental effect analyses, considering the significantly higher satisfaction rates and better economic sustainability in the MyCourse group, it would be preferable to offer the MyCourse group.

### Strengths and Limitations

An important strength of this study is that the evaluation was conducted in a real-world setting; recruitment was done through both offline and web-based channels, which could plausibly be used in case of future implementation. This study succeeded in recruiting cancer survivors from a range of cancer types. The median number of times participants logged into MyCourse was high (8 times). Several sensitivity analyses have attested to the robustness of the findings. The long-term follow-up of this study showed that AM is sustained over a long period. The results should be interpreted in light of the limitations of the study. Most of this study’s sample were women (86/103, 83.4%); thus, cautioning the generalization of the results to men. The participants were not blinded to their intervention allocation. Not all participants complied with the advised daily use of MyCourse for 4 weeks, and this might have influenced effects in the MyCourse group. A follow-up period of >12 months might be needed to find evidence of possible cost-utility of a digital AM intervention in cancer survivors.

### Conclusions

To the best of our knowledge, this is the first study on a digital AM intervention for cancer survivors, and it showed that alcohol use was reduced by one-third in both the MyCourse and control groups and that this effect was sustained over 12 months. No significant differential effect on alcohol use between the MyCourse group and the control group was observed at the follow-ups, although cancer survivors were more satisfied in the MyCourse group. From a societal perspective, the MyCourse group seems economically more sustainable for reducing the number of drinks, as a greater reduction in the number of drinks over time was observed against lower societal costs.

## Appendix

Multimedia Appendix 1 Additional information on Methods.

Multimedia Appendix 2 Attrition and satisfaction with the intervention.

Multimedia Appendix 3 Cost-effectiveness planes and cost-effectiveness acceptability curves after Winsorization.

Multimedia Appendix 4 CONSORT-eHEALTH checklist (V 1.6.1).

## Abbreviations

AM: alcohol moderation
AUDIT: Alcohol Use Disorders Identification Test
CEAC: cost-effectiveness acceptability curve
EQ-5D-5L: 5-level EuroQol 5 Dimension
ICER: incremental cost-effectiveness ratio
MCSDS: Marlowe-Crowne Social Desirability Scale
QALY: quality-adjusted life year
RCT: randomized controlled trial
SF-6D: Short Form 6-dimension
TLFB: Timeline Followback
ZUF-8: Patient Satisfaction Questionnaire
