# Supplementary material for: Effectiveness, Cost-effectiveness, and Cost-Utility of a Digital Alcohol Moderation Intervention for Cancer Survivors: Health Economic Evaluation and Outcomes of a Pragmatic Randomized Controlled Trial
Source: J Med Internet Res. 2022 Feb 1;24(2):e30095. doi: 10.2196/30095 (PMC8848232; doi:10.2196/30095)
Supplement: Multimedia Appendix 1 [file jmir_v24i2e30095_app1.docx]

Supplementary material for

“Cost-effectiveness of a digital alcohol moderation intervention for cancer survivors: health economic evaluation alongside a pragmatic randomized controlled trial”

Additional information on Methods

*Intervention*

The intervention could be used whenever the participants wanted to, for the duration of the study, but they were encouraged to login (almost) daily for at least four weeks. This encouragement happened for example through automated intervention reminder emails. The intervention also encouraged participants to engage their own social network through semi-automated email options throughout the program. Participants could for example share their moderation plan or answers to an exercise about high-risk situations for alcohol use. Questions about the intervention could be directed at the research staff through email or telephone, but for information on more intensive/guided AM support possibilities participants were referred to the national AM information line ([www.Alcoholinfo.nl](http://www.Alcoholinfo.nl)).

*Sensitivity Analyses*

Missing data for primary and secondary outcome measures were imputed using a second package: the Amelia 2-package. Inspection of the distributions of the imputed data showed that the mice-package predictive mean matching method outperformed the Amelia 2-package. This is not surprising as the Amelia 2-package can only handle multivariate normally distributed data, while alcohol use variables can be considered a form of count data and usually do not approach a multivariate normal distribution. We repeated the main analyses on both the Amelia 2-imputed data and the respondent only data (i.e., data without imputation).

For evaluation of number of drinks we also conducted a GLMM with log link function and a negative binomial distribution on the mice imputed data, a recommended approach for analysing substance use data (Atkins et al. 2013). For the incremental costs analyses we winsorized the most extreme healthcare costs at the 95^th^ percentile in a sensitivity analysis and based QALYs on SF6D scores instead of the EQ-5D-5L.
