# Supplementary material for: Effectiveness, Cost-effectiveness, and Cost-Utility of a Digital Alcohol Moderation Intervention for Cancer Survivors: Health Economic Evaluation and Outcomes of a Pragmatic Randomized Controlled Trial
Source: J Med Internet Res. 2022 Feb 1;24(2):e30095. doi: 10.2196/30095 (PMC8848232; doi:10.2196/30095)
Supplement: Multimedia Appendix 2 [file jmir_v24i2e30095_app2.docx]

Supplementary material for

“Cost-effectiveness of a digital alcohol moderation intervention for cancer survivors: health economic evaluation alongside a pragmatic randomized controlled trial”

Table 6. Attrition and satisfaction with the intervention

| **Measures** | | Primary and secondary measures | Total (N=103) | MyCourse (N=53) | Control (N=50) |
| --- | --- | --- | --- | --- | --- |
| **3 month follow-up** | | | | | |
|  | missing, n (%) |  |  |  |  |
|  | | number of drinks | 25 (24.3) | 10 (18.9) | 15 (30.0) |
|  | follow-up period in days, mean (SD) |  | 104.0 (15.9) | 101.9 (14.0) | 106.5 (17.9) |
|  | ZUF score, mean (SD) |  |  | 21.6 (4.5) | 17.7 (4.9) |
| **6 month follow-up** | | | | | |
|  | missing, n (%) |  |  |  |  |
|  | | number of drinks | 30 (29.1) | 13 (24.5) | 17 (34.0) |
|  | | AUDIT | 32 (31.1) | 13 (24.5) | 19 (38.0) |
|  | follow-up period in days, mean (SD) |  | 201.1 (33.6) | 197.5 (23.6) | 205.6 (43.2) |
| **12 month follow-up** | | | | | |
|  | missing, n (%) |  |  |  |  |
|  | | number of drinks | 30 (29.1) | 14 (26.4) | 16 (32.0) |
|  | | AUDIT | 31 (30.1) | 15 (28.3) | 16 (32.0) |
|  | follow-up period in days, mean (SD) |  | 375.7 (15.8) | 376.7 (18.2) | 374.6 (12.7) |
