# Supplementary material for: Effectiveness, Cost-effectiveness, and Cost-Utility of a Digital Alcohol Moderation Intervention for Cancer Survivors: Health Economic Evaluation and Outcomes of a Pragmatic Randomized Controlled Trial
Source: J Med Internet Res. 2022 Feb 1;24(2):e30095. doi: 10.2196/30095 (PMC8848232; doi:10.2196/30095)

Supplementary material for

“Effectiveness, cost-effectiveness and cost-utility of a digital alcohol moderation intervention for cancer survivors: health economic evaluation and outcomes of a pragmatic randomised controlled trial”

Figure Cost-effectiveness planes and cost-effectiveness acceptability curves after winsorization


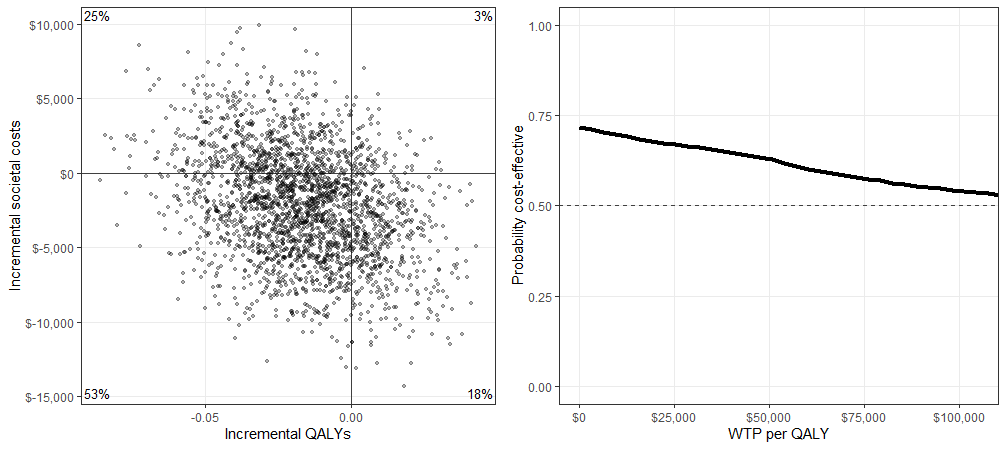


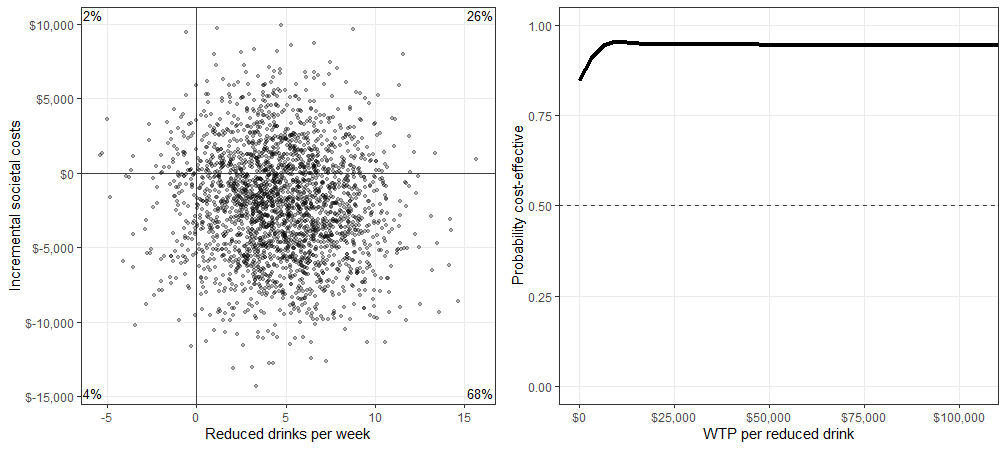

Supplement: Multimedia Appendix 3 [file jmir_v24i2e30095_app3.docx]
